# Synthesis of Nitronyl Nitroxide Radical-Modified Multi-Walled Carbon Nanotubes and Oxidative Desulfurization in Fuel

**DOI:** 10.3390/molecules29163896

**Published:** 2024-08-17

**Authors:** Min Tian, Haokang Huang, Gai Zhang, Haibo Wang

**Affiliations:** 1 School of Materials Science and Chemical Engineering, Xi’an Technological University, Xi’an 710021, China; tianmin@xatu.edu.cn (M.T.); huanghaokang@st.xatu.edu.cn (H.H.); 2 Department of Chemistry, School of Pharmacy, Air Force Medical University, Xi’an 710032, China

**Keywords:** carbon nanotubes, nitronyl nitroxide radical, thiophene, desulfurization, mechanism

## Abstract

Novel and highly stable nitronyl nitroxide radical (NIT) derivatives were synthesized and coated on the surface of multi-walled carbon nanotubes (MWCNTs) to improve their desulfurization performance. They were characterized by FTIR, UV-vis, SEM, XRD, Raman spectroscopy and ESR. Thiophene in fuel was desulfurized by molecular O_2_, and the oxidation activity of these compounds was evaluated. At a normal temperature and pressure, the degradation rates of thiophene by four compounds in 4 h can reach 92.66%, 96.38%, 93.25% and 89.49%, respectively. The MWCNTs/NIT-F have a high special activity for the degradation of thiophene, and their desulfurization activity can be recycled for five times without a significant reduction. The mechanistic studies of MWCNTs/NIT composites show that the ammonium oxide ion is the key active intermediate in catalytic oxidative desulfurization, which provides a new choice for fuel oxidative desulfurization. The results show that NIT significantly improves the photocatalytic performance of MWCNTs.

## 1. Introduction

Organic sulfide in fuel will produce a large amount of SOx gas when it is burned, causing serious harm to the ecological environment. First of all, SOx gas generated by combustion forms a strong acid solution when it encounters water, causing irreversible damage to relevant parts of the vehicle and property losses [1,2,3]. At the same time, there are certain risks in the driving process. In addition, SOx can lead to poisoning of catalysts in tail gas treatment devices, reduce catalytic activity, lead to substandard exhaust emissions, and increase the contents of COx, NOx and organic pollutants in the air [4]. At the same time, acid rain caused by SOx emissions will corrode all kinds of infrastructure, causing large economic losses. Hydrodesulfurization is a mainstream industrial desulfurization method [5], but it is difficult to efficiently remove thiophenes from diesel oil. However, based on the characteristics of the redox reaction, the more difficult the sulfides are to be hydroreduced, the easier they are to be oxidized. Therefore, oxidative desulphurization technology (ODS) with mild reaction conditions is more in line with the development trend of the green chemical industry [6,7]. Oxidative desulfurization mainly uses oxidants to gradually oxidize sulfides into smaller molecules under the catalysis of catalysts [8,9]. In the process of oxidative desulfurization, sulfone and sulfoxide are common products. The research on oxidative desulfurization is the selection and research and development of oxidants and catalysts. Common oxidants are hydrogen peroxide, ozone and oxygen [10,11]. At present, the direct selection of air has become the best choice to reduce costs and provide oxidants. Although the source of O_2_ in the air is abundant and the cost is low, its oxygen content is low and its oxidizing ability is poor. It is particularly important to use a desulfurization catalyst with an obvious effect and significant economic benefit that is non-toxic and pollution-free [12,13,14]. Green and pollution-free small-molecule organic oxidants may become a kind of desulfurization catalyst with better oxidation [15].

Multi-walled carbon nanotubes (MWCNTs) are one-dimensional carbon materials with a structured pore structure. Because of their good mechanical properties, unique thermal and electrical properties, strong specific surface area, they are called the “super fiber dimension” by scholars in this field and are widely used in the fields of adsorption catalytic desulfurization [16,17,18]. However, the strong interaction between MWCNTs makes them not easily dispersed and insoluble in any solvent, and their adsorption capacity is limited, which greatly restricts their applications [19,20,21]. It is very important to modify MWCNTs to improve their dispersion in solvent and substrate. A variety of modification methods for MWCNTs have been reported, and can be generally divided into physical modification and chemical modification [22,23]. Chemical modification can not only improve the dispersion of MWCNTs but also endow them with new properties. The most commonly used method is concentrated acid treatment in the field of chemical catalysis, which usually uses nitric acid and sulfuric acid to etch the surface of MWCNTs, resulting in defects on the surface of MWCNTs. These defects are often the junction sites of metal particles. Different types of functional groups, such as –COOH, –OH and –CHO, are inserted on the surface of MWCNTs according to the types of acids [24].

**Figure 1:**
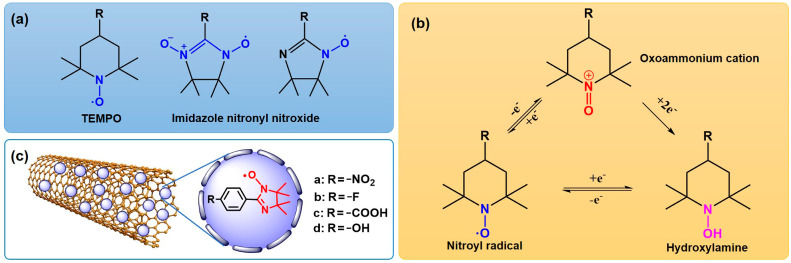
(**a**) The structures of TEMPO and NIT. (**b**) Redox transformation of the various oxidation states of NRs. (**c**) The structure of MWCNTs/NIT composites.

Nitronyl nitroxyl radicals (NRs) are a kind of stable small-molecule organic compounds containing C, N, O and a spin single electron that can be divided into two series: TEMPO (2,2,6,6-tetramethylpiperidine-N-oxyl) and imidazole nitronyl nitroxide radical (NIT) [25,26] (Figure 1a). TEMPO (4-hydroxy-2,2,6,6-tetramethylpiperidine-N-oxyl) has long been utilized as a biophysical tool in electron paramagnetic resonance (EPR) spectroscopic studies because of its stability and paramagnetic nature [27]. The easily modified chemical structure of NIT radicals is a key feature that makes obtaining a variety of NIT radicals with specialized structures easier. NRs are considered a good choice for the oxidation of organic compounds [28,29,30]. TEMPO is a valuable compound due to its reversible oxidation and reduction, low toxicity and stability. It can efficiently selectively catalyze the oxidation of alcohol to the corresponding aldehydes or ketones. Due to these advantages, TEMPO has gained widespread attention as a selective oxidation catalyst for alcohols [31,32,33,34] (Figure 1b). Anelli’s research group [35] first proposed a catalytic oxidation system composed of 4-methoxy-TEMPO and hypochlorite. The Matthew S. Sigman research group at the University of Utah, Scott J. Miller’s group at Yale University and Song Lin’s group at Cornell University have developed an unconventional catalyst optimization strategy by screening a set of substrate templates that include nitroxide radicals; Meso-diol was oxidized and desymmetrized with good enantioselectivity, and a series of chiral lactone compounds were constructed with high enantioselectivity [36]. The high activity of this system is particularly outstanding, which is incomparable to other catalytic systems. However, TEMPO has some drawbacks, such as being expensive and not recyclable, increasing production costs to some extent [37]. The recycling of TEMPO became a key issue for the system, and loading it into a nanosystem is an effective method to solve the problem of catalyst recycling.

The unique electronic properties of MWCNTs enable them to interact with the active components of catalysts, resulting in electron transfer and morphological changes that are conducive to the dispersion of active components and thus improve the catalytic activity [38]. In this paper, in order to improve the activity of MWCNTs, a new type of catalyst was prepared and coated on the surface of MWCNTs (Figure 1c). Using molecular oxygen as oxidant in simulated oil, the degradation performance of thiophene in crude oil was tested, and a reasonable catalytic desulfurization mechanism was proposed. The results indicated that these new catalysts have a high special activity for the degradation of thiophene, and its desulfurization activity can be recycled for 5 times without a significant reduction.

## 2. Results and Discussion

### 2.1. The Characteristic of MWCNTs/NIT Composites

**Figure 2:**
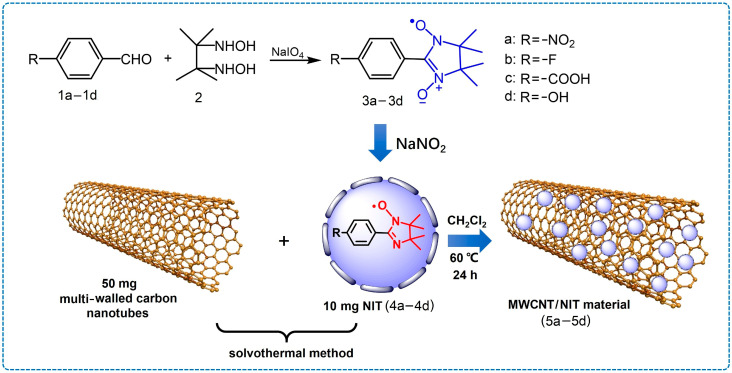
Formation process of MWCNTs/NIT composites.

The synthesis process of MWCNTs/NIT with different substituents is shown in Figure 2.

#### 2.1.1. ESR Spectra

**Figure 3:**
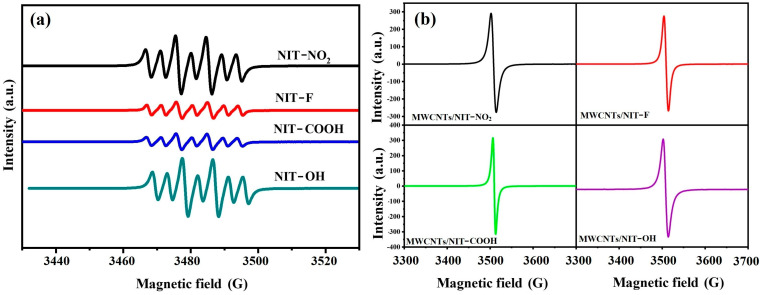
(**a**) ESR spectrum of NITs. (**b**) ESR spectrum of MWCNTs/NIT composites.

Due to its sensitivity and accuracy, ESR spectroscopy is the best tool for studying free radicals. As shown in Figure 3a, NIT derivatives, as small-molecule compounds, will produce seven fission peaks in DMF solution with a ratio of 1:1:2:1:2:1:1, indicating that these compounds contain a single electron. Figure 3b shows the electron paramagnetic resonance spectra of MWCNTs/NIT composites. Due to the poor solubility of the composite material, the use of solid powder samples did not produce fission peaks. It can be seen from the figure that the peak value appears in the range of 3500–3520, which is the characteristic peak of nitronyl nitroxide radicals, proving that MWCNTs/NIT composites have been successfully prepared.

#### 2.1.2. IR Spectra

**Figure 4:**
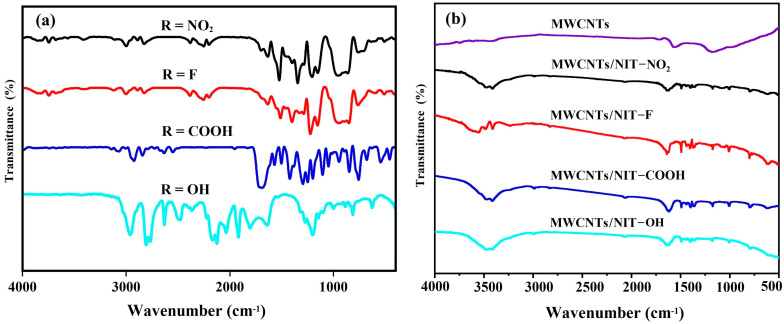
(**a**) FT-IR spectra of NITs. (**b**) FT-IR spectra of MWCNT/NIT composites.

The FT-IR spectra of the NIT radical derivatives **4a**–**4d** with different substituents are shown in Figure 4a. The stretching vibration peak of C–H on the aromatic ring appears between 3000–3150 cm^−1^. The C=C stretching vibration peaks of aromatic ring appear at 1589 cm^−1^, 1511 cm^−1^, 1566 cm^−1^ and 1584 cm^−1^, respectively. Generally, there are two characteristic peaks for nitronyl nitroxide radicals. The characteristic peak of N–O vibration absorption appears 1350 cm^−1^, while the peak near 1640 cm^−1^ corresponds to the stretching vibration of the stretching vibration absorption peak of C=N. For nitrobenzene formaldehyde nitronyl nitroxide radicals, the stretching vibration of -NO_2_ appears at 1600 cm^−1^. Evidently, for 4-carboxybenzaldehyde nitronyl nitroxide radicals, the characteristic peak at 1700 cm^−1^ is attributed to the C=O bond, and the peak at 1200 cm^−1^ corresponds to the stretching vibration of C–O in the carboxy benzaldehyde.

In the infrared spectrum of intermediate MWCNTs/NIT composites (Figure 4b), the peaks of different substituents appear at 1165 cm^−1^ and 1171 cm^−1^, which are the tensile vibration absorption peaks of N–O. The peaks 1488 cm^−1^, 1494 cm^−1^ and 1483 cm^−1^ belong to the absorption peak of C–N in the free radical part. Meanwhile, the peak at 1608 cm^−1^ is the absorption peak of C=N tensile vibration. The absorption peak generated by the carboxyl-substituted C=O double bond tensile vibration appears at 1635 cm^−1^. In addition, pure MWCNTs have four characteristic peaks: the peaks at 1551 cm^−1^ and 1159 cm^−1^ are attributed to the energy of carbon nanotube vibration, and peaks at 3414 cm^−1^ and 1704 cm^−1^ belong to the characteristic absorption peaks of graphene oxide. After recombination, the position of the peaks is shifted little, indicating that the carbon tube ring is not damaged by the introduction of free radicals. The results indicate that NIT is well recombined on MWCNTs.

#### 2.1.3. UV-Vis Spectra

**Figure 5:**
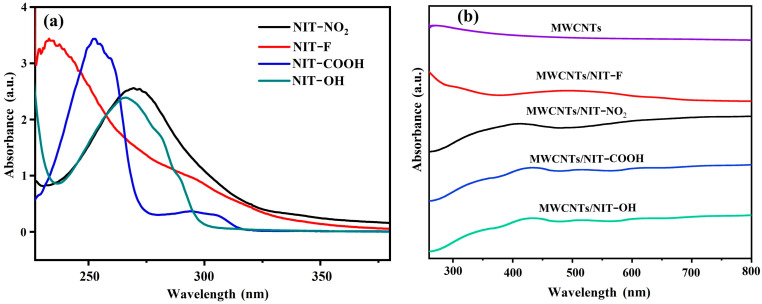
(**a**) UV-Vis spectra of NITs. (**b**) UV-Vis spectra of MWCNT/NIT composites.

The absorption characteristics of NIT radical derivatives corresponding to different substituents are different (Figure 5a), but their characteristic absorption peaks are concentrated at 220–350 nm, which is mainly the absorption produced by the conjugated system formed by the benzene ring and carbonyl group on the radical, such as the absorption peaks of chemical bonds such as C=O and C=N. In several complexes with different substituents of MWCNTs/NIT (Figure 5b), carbon nanotubes have no ultraviolet absorption, the π-π* transition of the characteristic band of O–N–C–N unit appears at the absorption peak return at 308–315 nm, which is longer than the wavelength of the individual chromophore, and its intensity is enhanced, indicating that the active site is still maintained after the combination of NIT and MWCNTs.

#### 2.1.4. XRD Spectrum

**Figure 6:**
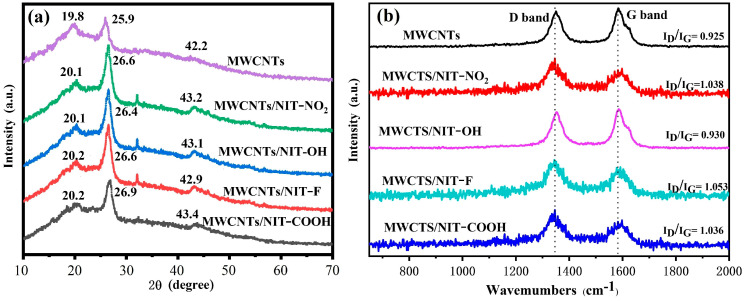
(**a**) XRD patterns of MWCNTs and MWCNTs/NIT composites. (**b**) Raman spectra of MWCNTs and MWCNTs/NIT composites.

The XRD patterns of MWCNTs, MWCNTs/NIT-NO_2_, MWCNTs/NIT-OH, MWCNTs/NIT-F and MWCNTs/NIT-COOH are shown in Figure 6a. Peaks at 19.8°, 25.9° and 42.2° indicate the interlaminar accumulation peaks (001), (002) and (101) and describe a well-developed layered structure of MWCNTs. Compared with pure MWCNTs, the XRD peaks of MWCNTs/NIT composites move slightly to a higher angle due to the formation of the NIT skeleton, with maximum values of 20.2°, 26.9° and 43.4° for MWCNTs/NIT-COOH. In addition, pure NIT is a completely amorphous liquid with no concrete crystal form. These results suggest that the introduction of NIT does not alter the structure of the original MWCNTs. 

#### 2.1.5. Raman Spectrum

It can be seen from the figure that all of the MWCNTs, MWCNTs/NIT-NO_2_, MWCNTs/NIT-OH, MWCNTs/NIT-F and MWCNTs/NIT-COOH have D peaks and G peaks at around 1350 cm^−1^ and 1580 cm^−1^ (Figure 6b). The D peak represents the absorption peak of sp^3^ hybrid carbon atoms and the defect and disorder of multi-walled carbon nanotubes. The higher the D peak, the higher the disorder of multi-walled carbon nanotubes. The G peak represents the graphitization degree of multi-walled carbon nanotubes, reflecting the symmetry and crystallization degree of the sp^2^ structure. Compared with MWCNTs, the positions of the D peak and G peak did not change much, and the relative strength of D peak and G peak (I_D_/I_G_) increased after the composite formed, indicating that the modification of nitronyl nitroxide radicals had a certain effect on the structure of MWCNTs, but the change was not significant. The results show that the introduction of nitronyl nitroxide radicals does not damage the structure of MWCNTs.

#### 2.1.6. Morphology

**Figure 7:**
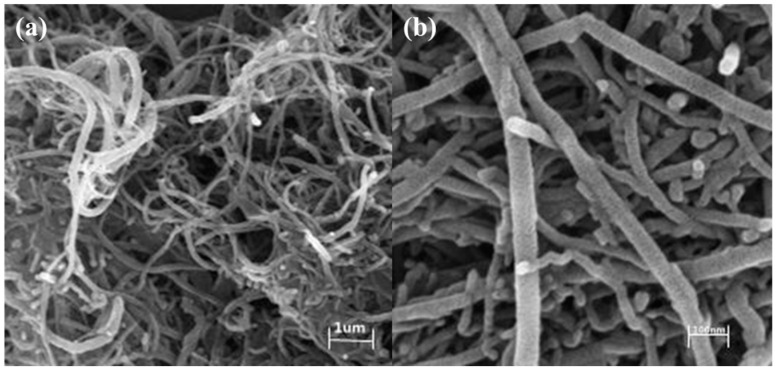
(**a**) SEM images of MWCNTs/NIT-F (1 μm). (**b**) SEM images of MWCNTs/NIT-F (100 nm)

The morphology of the prepared sample was further studied by SEM to determine its structural information. As shown in Figure 7, MWCNTs/NIT-F were examined at low multiples (1 μm) (Figure 7a). The MWCNTs were still visible, and the MWCNTs showed a relatively uniform staggered network structure. MWCNTs/NIT-F were detected in the high power range (100 nm) (Figure 7b). It was observed that the size of MWCNTs/NIT-F was less than 100 nm. The aggregate on the surface of the end cap increases and the diameter of the carbon tube increases, indicating that the nitronyl nitroxide radical-based carbon tube is successfully loaded.

### 2.2. Desulfurization Effects of NIT and MWCNT/NIT Composites

**Figure 8:**
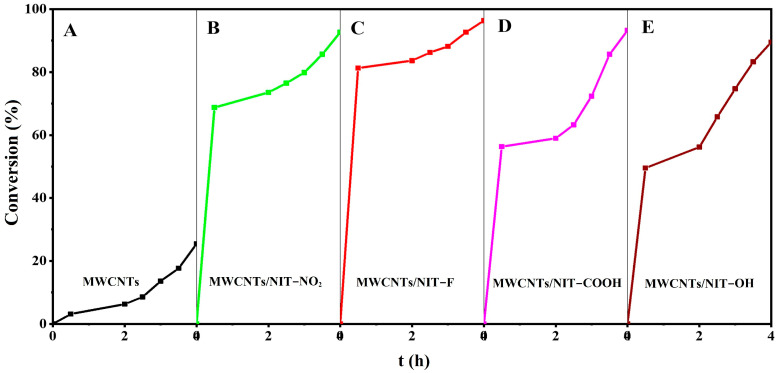
Comparison of the thiophene degradation rate of MWCNTs and MWCNT/NIT composites. A for MWCNTs; B for MWCNTs/NIT-NO_2_; C for MWCNTs/NIT-F; D for MWCNTs/NIT-COOH; E for MWCNTs/NIT-OH.

Figure 8A–E shows the temporal relationship between the catalytic degradation rate of thiophene and composites **5a**–**5d** with different substituents. The corresponding substituents were NO_2_, F, COOH and OH. After stirring in the dark at normal temperature and pressure for 4 h, the degradation rates of different substituents were 92.66%, 96.38%, 93.25% and 89.49%, respectively. In picture A, the adsorption degradation rate of carbon tube alone was 25.63%. The results show that **5a**–**5d** have good catalytic oxidation ability, and the introduction of free radicals can improve the degradation efficiency of carbon nanotubes. The order of the degradation activity of different substituents is MWCNTs/NIT-F > MWCNTs/NIT-COOH > MWCNTs/NIT-NO_2_ > MWCNTs/NIT-OH, mainly because the electron absorbing group makes it easier to expose single electrons, and the catalytic efficiency of the strong electronegative F (**5b**) is the highest, reaching 96.38%. Through the catalytic degradation of thiophene, it can be obtained that the activated MWCNTs with different substituents of nitronyl nitroxide radicals (NITs) have good degradation effect on thiophene; that is, they have a certain catalytic oxidation ability.

**Figure 9:**
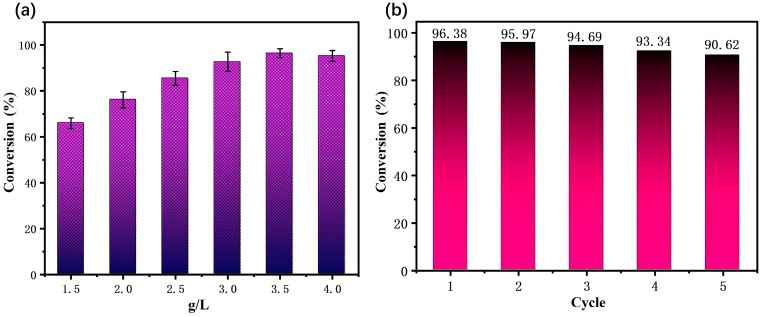
(**a**) Research on the optimal conditions for MWCNTs/NIT-F. (**b**) Stability test results for MWCNTs/NIT-F.

The amount of catalyst will directly affect the desulfurization effect. The effect of the catalyst dosage on thiophene transformation was studied by adding different catalyst dosages to 50 mL of model gasoline (2000 µL/L). As can be seen in Figure 9a, the conversion rate of thiophene gradually increased with the increase in the catalyst dosage. When the catalyst dosage of MWCNTs/NIT-F was 1.5, 2.0, 2.5, 3.0, 3.5 and 4.0 g/L, the conversion rates of thiophene were 65.88%, 76.13%, 85.44%, 92.65%, 96.38% and 95.23%, respectively. As the catalytic dosage increased to 3.5 g/L, the thiophene conversion reached a maximum value of 96.38%. Therefore, in this experiment, 3.5 g/L was selected as the optimal dosage of catalyst.

To test the repeated stability of the catalyst, the reusability of the MWCNTs/NIT-F composite was evaluated. The reactants were cooled to room temperature and filtered after the reaction, after which the catalyst was recovered via filtration, washed with acetonitrile and dried under a vacuum. This test was repeated five times. The results showed that the MWCNTs/NIT-F composite exhibited a 5.9% change during the five thiophene degradation cycles (Figure 9b). MWCNTs/NIT-F exhibited high stability in the repeated reaction.

### 2.3. Catalytic Degradation Mechanism of the MWCNT/NIT Composites

**Figure 10:**
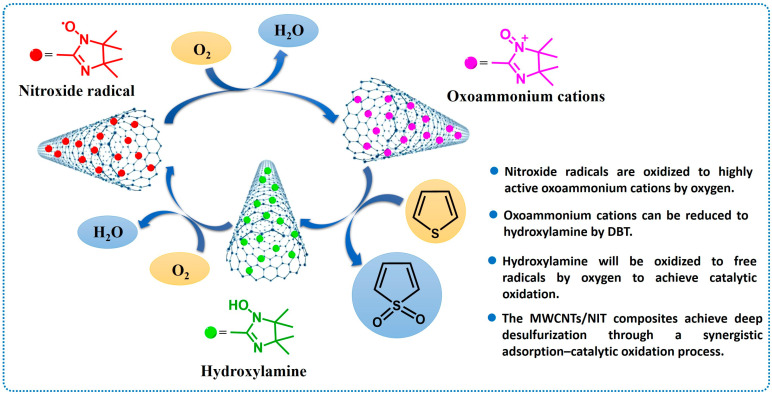
Catalytic degradation mechanism of MWCNTs/NIT composites.

By summarizing the experimental results for the photocatalytic degradation of thiophene in thiophene by MWCNTs/NIT composites, the mechanism of oxidative catalytic desulfurization was proposed (Figure 10): MWCNTs and nitronyl nitroxide radicals (NITs) could achieve the simultaneous adsorption and catalytic oxidative degradation of thiophene based on two parts of the host. First, thiophene molecules to be degraded were dissolved in cyclohexane solution. The MWCNTs/NIT composites serving as the catalyst were fully exposed to thiophene. When the oxygen was passed, NIT was oxidized to the highly active oxoammonium cations, the exposed oxoammonium cations oxidized thiophene that contacted them in solution because of their high oxidation activity, and these cations are reduced to hydroxylamine. Then, hydroxylamine will be oxidized to free radicals by oxygen to achieve catalytic oxidation. On the other hand, part of the thiophene was adsorbed on the MWCNTs, and a part of the adsorbed thiophene was catalyzed and degraded by the NITs on the MWCNTs in the presence of oxygen. The MWCNTs/NIT composites achieved deep desulfurization through a synergistic adsorption–catalytic oxidation process.

**Figure 11:**
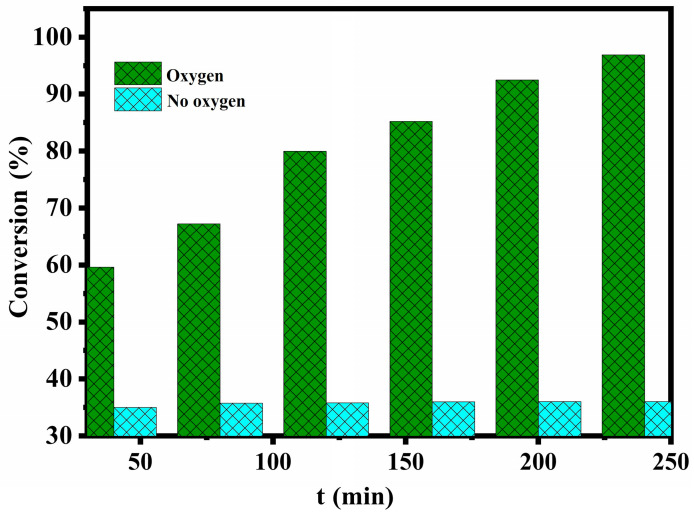
Influence of the oxygen supply on the desulfurization efficiency.

In order to study the role of molecular oxygen in catalyst desulfurization, two sets of experiments were set up. In the absence of oxygen, the desulphurization rate of the MWCNTs/NIT-F composite remained at about 36%, and when air was injected into the flask with an air pump, the desulphurization rate reached 96.38% after 4 h (Figure 11). The main reason is that when there is no oxygen, the free radicals supported by the MWCNTs can only partially oxidize thiophene and convert it to hydroxylamine, which is mainly the adsorption and degradation of the carbon tube. When air is continuously injected into the flask, the oxygen in air can convert hydroxylamine to the original active free radical through the oxoammonium cation. Therefore, the NIT nitronyl nitroxide radical realizes the recovery of single electron activity, continues to catalyze the oxidation of thiophene in the system, and realizes continuous desulfurization. Finally, thiophene is adsorbed and converted into sulfate ions and sulfones, completing the adsorption–catalytic oxidation process.

## 3. Materials and Methods

### 3.1. Experimental Reagents and Instruments

2,3-Dis(hydroxylamino)-2,3-dimethylbutane was self-made according to the literature method [39], and substituted benzaldehyde (Aladdin Chemical Reagent Co., Ltd., Shanghai, China), thiophene (Chengdu Kailong Chemical Reagent Co., Ltd., Chengdu, China), oxidized MWCNTs (Suzhou Tanfeng Graphene Technology Co., Ltd., Suzhou, China), and all other reagents were analytical reagent grade and were used without further purification.

For the infrared test, a solution of the same concentration of free radicals (dichloride as the solvent) was added to a KBr sheet. After drying, the TENSOR II infrared spectrometer with a scanning range of 4000–500 cm^−1^ was used for the analysis. Ultraviolet-visible spectroscopy is based on dichloride as the solvent and the characteristic absorption peak of the substance was tested using the UV-2550 instrument produced by Shimazu Company. The scanning range was 200–800 nm. Electron paramagnetic resonance spectroscopy was mainly used to test the single electron contained in the structure through electron spin. In this experiment, the EPR spectra of nitronyl nitroxide radicals (NITs) were detected and analyzed at room temperature on an ESR5000 produced in Brouck, Germany, using DMF as solvent, and the composite material was tested as a solid powder sample. An X-ray diffractometer is used to analyze and test the target product using the D2 PHASER X-ray diffractometer produced by Brock AXS at a scanning speed of 4°/min, with a scanning angle range of 5° to 50°; SEM images were taken with QuantaFEG 400 field emission scanning electron microscope at different magnifications. The scanning range of nitronyl nitroxide radicals (NITs) and MWCNTs/NIT composites was 3500–600 cm^−1^ using the confocal micro Raman spectroscope from Lab RAM HR Evolution in France and a 532 nm solid-state laser source.

### 3.2. Synthesis of Nitronyl Nitroxide Radical Derivatives 

The nitronyl nitroxide radicals **1a**–**1d** were synthesized as shown in Figure 2. A total of 2.5 mmol of substituted benzaldehyde and 2.0 mmol (300 mg) 2,3-dis(hydroxylamino)-2,3-dimethylbutane were dissolved in 40 mL of newly distilled methanol and refluxed for 24 h. A yellow solution and a lot of white solid on the bottle wall were obtained. The methanol was removed when the reaction was complete. Then, the white solid product was suspended in 40 mL of methylene chloride, and 530 mg of a NaIO_4_ aqueous solution (2.5 mmol of NaIO_4_ in 20 mL of water) was slowly dropped into the mixture at 0 °C and stirred for 10 min. The organic layer divided from the mixture was dried with anhydrous Na_2_SO_4_. The crude product was purified by column chromatography on silica gel using ether/petroleum/dichloromethane (4:2:1) as the eluent, and a dark blue solid product was obtained. A total of 0.3 mmol of the remaining substance was dissolved in 20 mL of dichloromethane, and sodium nitrite (172 mg, 2.5 mmol) was added and refluxed for 1 h. The crude product was purified by column chromatography on silica gel using ether/petroleum/dichloromethane (4:2:1) as the eluent, and a yellow solid product was obtained.

Nitro-substituted benzaldehyde radical (**4a**) 0.054 g (68.63%), red solid; MS (*m*/*z*): 264.1351 (MNa^+^); IR (KBr) cm^−1^: 3014, 2236, 1638, 1600, 1589, 1337, 1197, 917, 834; ESR (CH_2_Cl_2_): g factor, 2.0016; a_N_ (G), 4.23G. Anal. Cald. for C_13_H_16_N_3_O_3_: C, 59.53; H, 6.15; N, 16.02%. Found: C, 59.57; H, 6.16; N, 15.99%.

Fluoro-substituted benzaldehyde radical (**4b**) 0.056 g (79.60%), yellow solid; MS (*m*/*z*): 237.1404 (MNa^+^); IR(KBr) cm^−1^: 3179, 2777, 1890, 1642, 1511, 1446, 1380, 884; ESR (CH_2_Cl_2_): g factor, 2.0016; a_N_ (G), 4.23G. Anal. Cald. for C_13_H_16_N_2_OF: C, 66.36; H, 6.85; N, 11.91%. Found: C, 66.32; H, 6.88; N, 11.94%.

Carboxyl-substituted benzaldehyde radical (**4c**) 0.042 g (53.57%), yellow solid; MS (*m*/*z*): 263.1391 (MNa^+^); IR (KBr) cm^−1^: 3057, 2923, 1708, 1646, 1566, 1369, 1288, 1185, 838, 753; ESR (CH_2_Cl_2_): g factor, 2.0016; a_N_ (G), 4.23G. Anal. Cald. for C_14_H_17_N_2_O_3_: C, 64.35; H, 6.56; N, 10.72%. Found: C, 64.42; H, 6.52; N, 10.56%.

Hydroxyl-substituted benzaldehyde radical (**4d**) 0.036 g (51.50%), red solid; MS (*m*/*z*): 231.1696 (MNa^+^); IR (KBr) cm^−1^: 3401, 3185, 2765, 1914, 1774, 1635, 1584, 1349, 841; ESR (CH_2_Cl_2_): g factor, 2.0016; a_N_ (G), 4.23 G. Anal. Cald. for C_13_H_17_N_2_O_2_: C, 66.93; H, 7.35; N, 12.01%. Found: C, 66.89; H, 7.38; N, 12.05%.

### 3.3. Synthesis of MWCNTs/NIT Composites (Nitroxide Radical-Modified Carbon Nanotubes)

In this experiment, MWCNTs/NIT composites with different substituents were synthesized by the solvothermal method. In total, 10 mg of NIT-Ph-R, 50 mg of MWCNTs and 10–20 mL of dichloromethane was placed in a high-pressure reactor, reacted at 60 °C for 24 h, centrifuged at 8000 RPM for 10 min, then washed twice with deionized water and the MWCNTs/NIT materials (**5a**–**5d**) were dried.

### 3.4. Evaluation of Catalytic Activity

A total of 1 mL of thiophene was added to 499 mL of cyclohexane to prepare 500 mL of 2000 µL/L model gasoline. A total of 0.18 g of MWCNTs/NIT composites and 50 mL of model gasoline (2000 µL/L) were added to a three-way flask and stirred at normal temperature and pressure without illumination. At the same time, an air pump was used to continuously inject air and samples were taken every 40 min. The samples were centrifuged at 3000 r/min for 5 min, and the supernatant was absorbed at 1 µL with a chromatography sampler. The absorbance of each sample was detected by gas chromatography, and the average value of each sample was measured three times. The desulfurization rate was calculated with the following formula: W% = (C_0_ − C_t_)/C_0_ × 100%, where C_0_ is the initial concentration of sulfur, and C_t_ is the concentration of sulfur after a period of desulfurization.

### 3.5. Evaluation of Catalyst Recycling

The repeated recovery process of the catalyst was as follows: the system reaction after the last desulfurization was cooled to room temperature and diethyl ether was added to precipitate the desulfurization catalyst fully. The precipitated catalyst was filtered and then the catalyst was washed with distilled water three times and dried under vacuum.

## 4. Conclusions

In this paper, four kinds of MWCNTs/NIT composites with different substituents were designed, prepared and characterized by FTIR, UV-vis, ESR, SEM, XRD and Raman spectroscopy. Then, the desulfurization performance of thiophene in model fuel containing cyclohexane was tested under normal temperature, atmospheric pressure and air conditions. Under the optimal reaction conditions, all four MWCNTs/NIT composites could achieve deep desulfurization after 4 h. The desulfurization rate of MWCNTs/NIT-F reached 96.38%, and a cyclic desulphurization test was carried out on it. The experiment showed that the catalyst still had a high desulfurization rate after five regeneration cycles, and the desulfurization rate reached 90.62%, indicating that the catalyst had good cyclic stability. At the same time, the mechanism of MWCNTs/NIT composites with adsorption and catalytic oxidation desulfurization was studied. The mechanism shows that nitronyl nitroxide radicals (NITs) fixed on MWCNTs can improve the catalytic activity of MWCNTs so as to realize the collaborative catalytic oxidation of MWCNTs and nitronyl nitroxide radicals (NITs) to remove thiophene, which provides an experimental basis for exploring and synthesizing new modified MWCNTs as desulfurization catalysts.

## Data Availability

The source data for the underlying tables and figures are available from the authors upon request.
